# Uncovering the neurochemistry of reward and aversiveness

**DOI:** 10.3389/fnmol.2013.00041

**Published:** 2013-11-19

**Authors:** Carmelo M. Vicario

**Affiliations:** School of Psychology, University of QueenslandBrisbane, QLD, Australia

**Keywords:** dopamine, serotonin, reward, aversiveness, neurochemistry

In a recent issue of Science was published an interesting report regarding the role of dopaminergic neurons on reward and aversiveness (Fiorillo, 2013). In contrast to what is claimed by some physiological and computational models (e.g., Schultz et al., 1997), the author provides a number of arguments to support the suggestion that reward and punishment have to be considered as two distinct neural dimensions. In particular, the author focuses on the evidence that dopaminergic neurons are selectively activated by rewarding outcomes, while these same neurons are insensitive to aversiveness. Thus, the author concludes a selective role for dopamine in detecting reward, and suggests that other (not dopaminergic) neurons should be sensitive to aversiveness. Recent insights suggest that these neurons may be serotonergic. For example, Limebeer et al. (2004) have shown that depletion of forebrain serotonin (5-HT) by 5,7 dihydroxytryptamine (5,7-DHT) lesions prevents induced conditioned disgust reactions such as “gaping,” the predominant conditioned rejection reaction (Parker, 2003) to “taste-related” aversive stimulation. Wright et al. (2010) have shown that learning to avoid odors associated with the malaise caused by ingesting toxins is mediated by serotonin. Moreover, it was recently demonstrated that partial depletion of 5-HT, in the insular cortex, prevents induced conditioned disgust reactions (Tuerke et al., 2012; see also Vicario, 2013, in press for a recent discussion about this issue). Finally, there is evidence of altered serotoninergic activity in Anorexia Nervosa (AN) (e.g., Jean et al., 2012), a psychiatric disorder characterized by a marked aversive sensitivity for food (Vicario, 2013, in press).

The works mentioned above support the “two dimensions” hypothesis (Fiorillo, 2013) for reward and aversiveness. In particular, it is suggested that serotoninergic neurons could be the principal candidate of aversiveness processing.

The evidence provided by these studies, which are based on the behavioral effects of block (removal) of serotonin, are difficult to interpret given the relevant number of variables that remains out of control. Thus, the definitive evidence could come from experiments that monitor activity of serotonin neurons on a fast timescale and relate it to reward and aversiveness in carefully controlled experiments. Moreover, future works might wish to investigate the existence of “ON” or “OFF” operating mechanism also for serotonin. In fact, if serotonin is the key neurotransmitter underlying aversiveness, there may be some serotonin neurons that signal aversive-ON (punishment) and others that signal aversive-OFF (absence of punishment and “negative reinforcement”).
